# Effect of Animal and Industrial *Trans* Fatty Acids on HDL and LDL Cholesterol Levels in Humans – A Quantitative Review

**DOI:** 10.1371/journal.pone.0009434

**Published:** 2010-03-02

**Authors:** Ingeborg A. Brouwer, Anne J. Wanders, Martijn B. Katan

**Affiliations:** 1 Department of Health Sciences, Faculty of Earth and Life Sciences, EMGO Institute for Health Care Research, VU University, Amsterdam, The Netherlands; 2 Division of Human Nutrition, Wageningen University, Wageningen, The Netherlands; Leiden University Medical Center, Netherlands

## Abstract

**Background:**

*Trans* fatty acids are produced either by industrial hydrogenation or by biohydrogenation in the rumens of cows and sheep. Industrial *trans* fatty acids lower HDL cholesterol, raise LDL cholesterol, and increase the risk of coronary heart disease. The effects of conjugated linoleic acid and *trans* fatty acids from ruminant animals are less clear. We reviewed the literature, estimated the effects *trans* fatty acids from ruminant sources and of conjugated *trans* linoleic acid (CLA) on blood lipoproteins, and compared these with industrial trans fatty acids.

**Methodology/Principal Findings:**

We searched Medline and scanned reference lists for intervention trials that reported effects of industrial trans fatty acids, ruminant trans fatty acids or conjugated linoleic acid on LDL and HDL cholesterol in humans. The 39 studies that met our criteria provided results of 29 treatments with industrial *trans* fatty acids, 6 with ruminant *trans* fatty acids and 17 with CLA. Control treatments differed between studies; to enable comparison between studies we recalculated for each study what the effect of *trans* fatty acids on lipoprotein would be if they isocalorically replaced *cis* mono unsaturated fatty acids. In linear regression analysis the plasma LDL to HDL cholesterol ratio increased by 0.055 (95%CI 0.044–0.066) for each % of dietary energy from industrial *trans* fatty acids replacing *cis* monounsaturated fatty acids The increase in the LDL to HDL ratio for each % of energy was 0.038 (95%CI 0.012–0.065) for ruminant trans fatty acids, and 0.043 (95% CI 0.012–0.074) for conjugated linoleic acid (p = 0.99 for difference between CLA and industrial *trans* fatty acids; p = 0.37 for ruminant versus industrial *trans* fatty acids).

**Conclusions/Significance:**

Published data suggest that all fatty acids with a double bond in the *trans* configuration raise the ratio of plasma LDL to HDL cholesterol.

## Introduction

*Trans* fatty acids arise either from industrial hydrogenation, or from biohydrogenation in ruminant animals. Artificial *trans* fatty acids are produced by partial hydrogenation of vegetable or fish oils with hydrogen gas and a metal catalyst. Consumption of such industrial *trans* fatty acids raises the total to HDL cholesterol ratio in blood and the risk of coronary heart disease [1]–[5]. Natural *trans* fatty acids are produced in the rumens of cows and sheep. They arise through partial hydrogenation and/or isomerization of *cis*-unsaturated fatty acids from the feed by hydrogen produced during oxidation of substrates, with bacterial enzymes as catalysts. As a result the fat in milk, butter, cheese and beef contains 2–9% *trans* fatty acids [6]–[8]. Because of the steep reduction in the production and intake of industrial *trans* fatty acids, ruminant fats are now the major source of *trans* fatty acids in most European countries [9] and will likely become so in the USA [10].

The effects of ruminant *trans* fatty acids on lipoproteins and heart disease are unclear. Some epidemiological studies showed no association [1], [3], [11] between ruminant *trans* fatty acid intake and heart disease risk, one showed a non-significant inverse association [2] and one a non-significant positive association [4]. Data on the effects of ruminant *trans* fatty acids on plasma lipoproteins in humans are limited. One study found adverse effects of high intakes, but not of low intakes of ruminant *trans* fatty acids [12]. Another study suggested that ruminant *trans* fatty acids produce higher LDL and HDL cholesterol levels than industrial *trans* fatty acids in women, but not in men [13].

**Figure 1:**
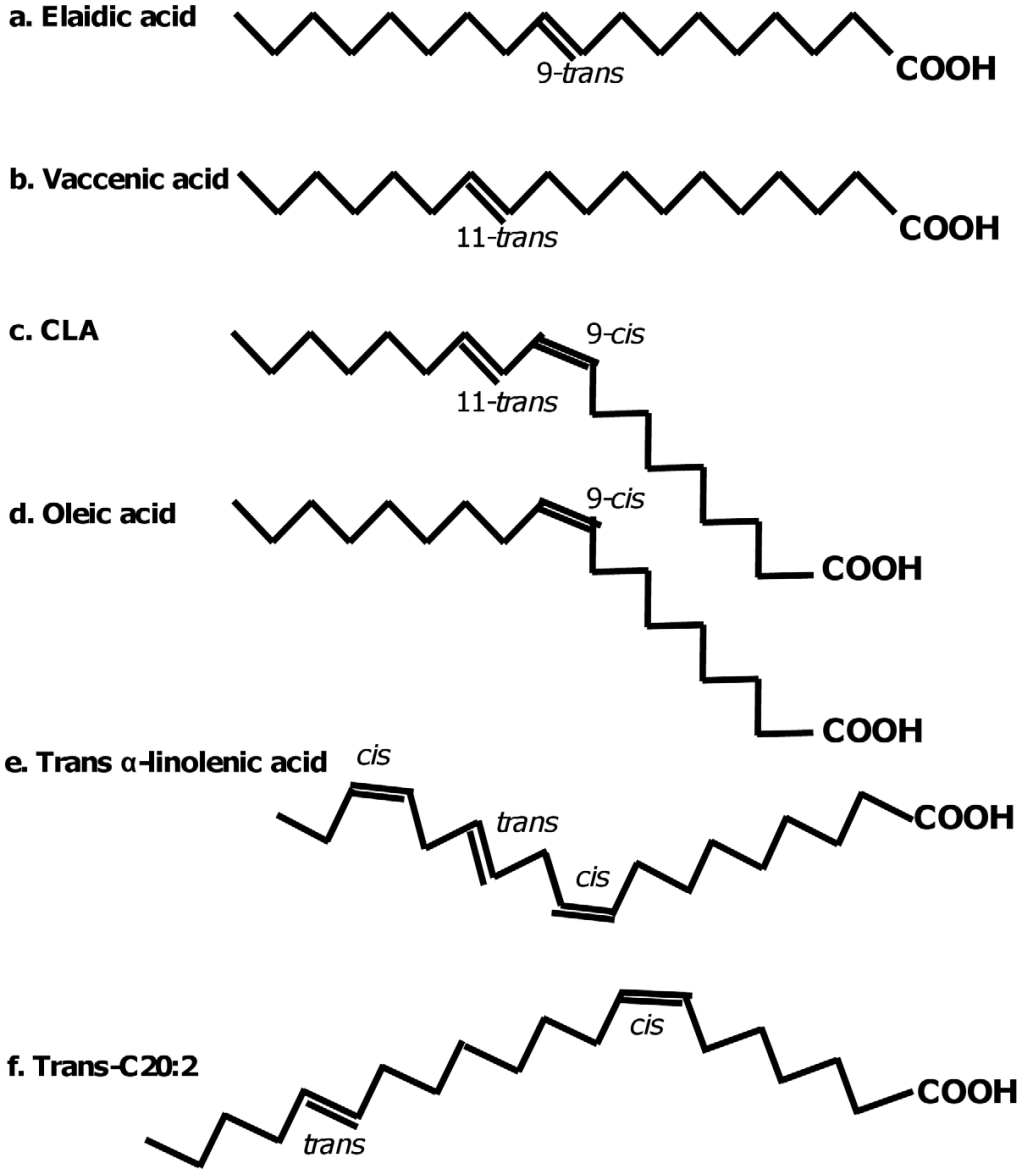
Structures of *cis*- and *trans* fatty acids. Elaidic acid (9-*trans*-C18:1) is a typical industrial trans fatty acid, produced by partial hydrogenation of vegetable oil. Vaccenic acid (11-*trans*-C18:1) is the predominant trans fatty acid in milk and meat from ruminant animals, although small amounts are also found in industrially hydrogenated fats. The 9,11 isomer of conjugated linoleic acid or CLA (9-cis, 11-*trans*-C18:2) is found almost exclusively in ruminant fat; industrial production of CLA yields a mixture of 9,11 and 10,12 isomers. Oleic acid (9-cis -C18:1) is the predominant cis-unsaturated fatty acid in the diet. The location of the trans bond in trans isomers of alpha-linolenic acid is not known precisely; for this figure it has been assigned arbitrarily to the 6 location. The same holds for the trans bonds in the trans isomers of C20:1, C20:2, C22:1 and C22:2 that arise from eicosapentaenoic acid (EPA) and docosahexaenoic acid (DHA) during partial hydrogenation of fish oil.

Industrial and ruminant fats contain similar species of *trans* fatty acids, but in different proportions (figure 1). Industrial trans fatty acids come in two kinds: partially hardened vegetable oils mainly contain *trans* isomers of oleic acid (figure 1a), the major one being C18:1 *trans*-9 or elaidic acid (figure 1d) and C18: 1 *trans*-10. Partially hydrogenated fish oils mainly contain *trans* isomers of C20:1, 20:2, 22:1 and 22:2 (figure 1f). Partially hydrogenated vegetable oils also contain smaller amounts of C18: 1 *trans*-8, and C18:1 *trans*-11 or vaccenic acid (figure 1b), and *trans* isomers of alpha-linolenic acid may arise during deep-fat frying. All these industrial *trans* fatty acids raise the LDL to HDL cholesterol ratio [5], [14]–[16].

In milk and meat C18:1 *trans*-11 (vaccenic acid) is the predominant *trans* fatty acid. In addition, ruminant fats contain small amounts of *cis*-9, *trans*-11 18:2 (conjugated linoleic acid, abbreviated to CLA in this paper unless otherwise mentioned; figure 1c). *Cis*-9, *trans*-11 18:2 CLA is also formed from ingested vaccenic acid in animals and in humans [17]. CLA is also widely sold as a supplement in the form of capsules. Most CLA capsules contain a mix of *cis*-9, *trans*-11 CLA and another CLA isomer *trans*-10, *cis*-12 CLA. These CLA-preparations are promoted for weight loss, although studies in humans have been inconclusive on this aspect [18], [19].

Countries such as Denmark that have banned the use of *trans* fatty acid in foods have excluded ruminant trans fatty acids. The US Food and Drug Administration includes ruminant *trans* fats in its labeling rules for *trans* fatty acids, but exempts CLA.

As the effects of the natural *trans* fatty acids are unclear we here review effects of different *trans* fatty acids on lipoprotein levels in human intervention trials.

## Methods

We review randomized intervention trials that investigated effects of either industrial *trans* fatty acids, or conjugated linoleic acid, or other ruminant *trans* fatty acids on the LDL to HDL cholesterol ratio, and on LDL and HDL cholesterol concentrations.

### Selection of Studies

We searched Medline for all relevant original-research papers published in English between January 1990 and January 2010 using as search terms: “(trans fat OR trans fatty acids, OR CLA) AND LDL”. We limited our search to human studies. We also scanned reference lists to ensure completeness.

We selected dietary trials that reported effects of industrial trans fatty acids, CLA or other ruminant trans fatty acids on LDL and HDL cholesterol levels. We also included studies that reported effects of CLA supplements. Such supplements usually contain a mix of *cis*-9, *trans*-11 CLA, the same conjugated linoleic acid as in ruminant fats, and *trans*-10, *cis*-12 CLA.

Studies had to have a parallel, crossover, or Latin-square design. We excluded before-and-after (sequential) designs that lacked a control or comparison group or period. Treatment periods had to be at least 13 days as that is the minimum period to achieve a new steady-state concentration of plasma lipoproteins [20], [21]. Trials in which subjects lost or gained significant amounts of weight were excluded [22], [23] as this has an effect on blood lipoproteins independent of dietary composition [24], [25].

### Statistical Analysis

Some studies compared *trans* fatty acids with saturated fatty acids, or compared two sources of *trans* fatty acids only. We recalculated these results to effects relative to isocaloric amounts of *cis* mono-unsaturated fatty acids according to the equations of Mensink et al. [26]. To maintain uniformity, we recalculated the ratio of LDL to HDL cholesterol from mean LDL and HDL levels for all studies, even where ratios had been reported. Data from different studies were combined using linear regression analysis with intake of *trans* fatty acids as independent and change in the plasma LDL to HDL cholesterol ratio, LDL cholesterol and HDL cholesterol as dependent variables. We did not weigh studies by number of subjects or standard error of the estimate because the LDL to HDL ratios are calculated on the basis of mean values of treatment differences within studies without an estimate of variation. Regression lines were forced through the origin because a zero change in diet should produce a zero change in blood lipids. We also tested logarithmic models in order to check whether our assumption of a linear dose-response relation was appropriate.

All studies of CLA except one [27] used CLA supplements on top of uncontrolled ad-lib diets. Doses of CLA in these studies were reported as grams per day. In order to recalculate these to percent of energy we assumed an energy intake of 2250 kcal/day, because a usual energy intake is 2500 kcal per day for men and 2000 kcal per day for women, and most studies enrolled approximately equal numbers of men and women.

## Results

**Figure 2:**
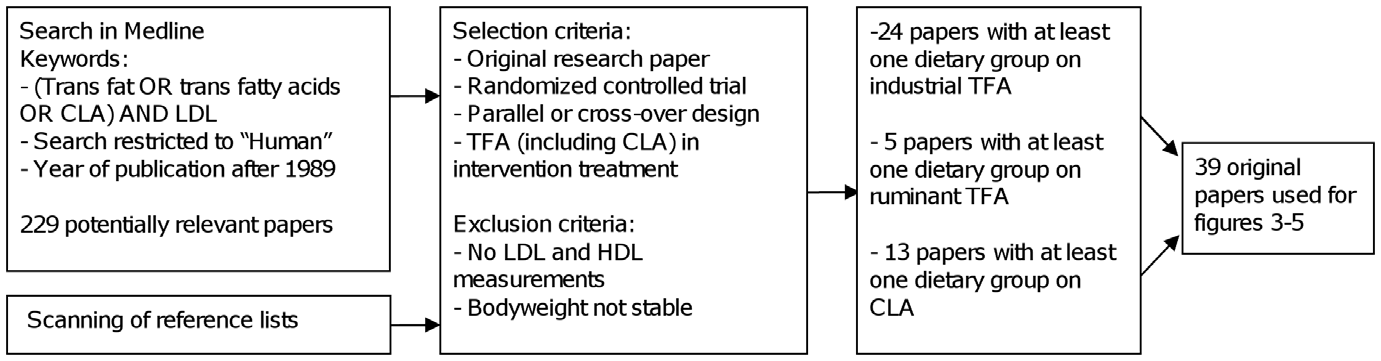
Flow chart of a search details for trials included in figure 3. TFA: trans fatty acids.

Figure 2 shows a flow diagram of the selection of studies for this review. Table 1 provides an overview of the studies and their outcomes for the LDL to HDL cholesterol ratio. For industrial *trans* fatty acids we started out with the data of Ascherio et al. [28] and extended these with results of studies on industrial or ruminant trans fatty acids published since [10], [12]–. There were 23 studies with controlled diets in which the fat in the diet was provided by the investigators, so-called dietary controlled studies on industrial *trans* fatty acids [12]–[15], [27], [30]–[37], [40]–[49], 5 dietary controlled studies on ruminant *trans* fatty acids [12], [13], [29], [38], [39], and 1 CLA study with dietary control [27] and 12 studies on CLA supplements in which the diet was not controlled [50]–[61]. We excluded two studies in which the subjects lost significant amounts of weight [22], [23] as this is known to influence lipoprotein levels. We also excluded one study which used a sequential design [62] and one small Malaysian study which showed a discordant, extremely strong adverse effect, of industrial *trans* fatty acids [63].

**Table 1:** Randomized trials assessing the effect on the ratio of LDL to HDL cholesterol of industrial or ruminant trans fatty acids or CLA, relative to *cis* unsaturated fatty acids.

Trial	Design	Tested fat	N (men/women)	Duration of treatment	Diet / Supplement	Randomisation and stratification	Drop out Rate (%)	Dose (delta en% trans fat)	Difference in LDL/HDL
Mensink and Katan, 1990	Cross-over	Industrial	59 (25/34)	21 d	Diet	Random order stratified by sex and OC use	0	10.9	0.55
Zock and Katan, 1992	Cross-over	Industrial	56 (26/30)	21 d	Diet	Random order stratified by sex	3.4	7.6	0.32
Nestel et al. 1992	Cross-over	Industrial	27 (27/0)	3 wk	Diet	Control period fixed / interventions random order	0	4.3	0.37
Lichtenstein et al. 1993	Cross-over	Industrial	14 (6/8)	28 d	Diet	Random order	6.7	3.8	0.30
Judd et al. 1994	Cross-over	Industrial	58 (29/29)	42 d	Diet	Random order stratified by sex and cholesterol	9.4	3.0	0.18
Judd et al. 1994	Cross-over	Industrial	58 (29/29)	42 d	Diet	Random order stratified by sex and cholesterol	9.4	5.7	0.26
Almendingen et al. 1995	Cross-over	Industrial	31 (31/0)	21 d	Diet	Random order	6.1	7.6	0.08
Almendingen et al. 1995	Cross-over	Industrial	31 (31/0)	21 d	Diet	Random order	6.1	7.1	0.57
Aro et al. 1997	Parallel	Industrial	80 (31/49)	35 d	Diet	Random, matched by cholesterol	0	8.3	0.41
Sundram et al. 1997	Cross-over	Industrial	29 (20/9)	4 wk	Diet	Random order	6.9	5.5	0.75
Muller et al. 1998	Cross-over	Industrial	27 (0./27)	17 d	Diet	Random order	10.0	6.8	0.36
Muller et al. 1998	Cross-over	Industrial	16 (0/16)	14 d	Diet	Random order	0	6.6	0.33
Lichtenstein et al. 1999	Cross-over	Industrial	36 (18/18)	35 d	Diet	Random order	0	0.4	0.03
Lichtenstein et al. 1999	Cross-over	Industrial	36 (18/18)	35 d	Diet	Random order	0	2.8	0.12
Lichtenstein et al. 1999	Cross-over	Industrial	36 (18/18)	35 d	Diet	Random order	0	3.6	0.23
Lichtenstein et al. 1999	Cross-over	Industrial	36 (18/18)	35 d	Diet	Random order	0	6.2	0.40
Louheranta et al. 1999	Cross-over	Industrial	14 (0/14)	4 wk	Diet	Random order	6.7	5.1	0.17
De Roos et al. 2001	Cross-over	Industrial	32 (11/21)	4 wk	Diet	Random order	0	9.0	0.67
Judd et al. 2002	Cross-over	Industrial	50 (50/0)	35 d	Diet	Random stratified by BMI and LDL	7.4	8.2	0.52
French et al. 2002	Cross-over	Industrial	10 (0/10)	30 d	Diet	Not described	0	5.6	0.59
Han et al. 2002	Cross-over	Industrial	19 (8/11)	32 d	Diet	Random order	0	6.1	0.64
Lovejoy et al. 2002	Cross-over	Industrial	25 (12/13)	28 d	Diet	Random order	19.4	7.3	0.07
Dyerberg et al. 2004	Parallel	Industrial	79 (79/0)	8 wk	Diet	Random (envelop with code)	9.2	5.9	0.29
Lichtenstein et al. 2006	Cross-over	Industrial	30 (14/16)	5 wk	Diet	Random order	28.6	1.9	0.20
Vega-Lopez et al. 2006	Cross-over	Industrial	15 (5/10)	5 wk	Diet	Random order	0	3.6	0.42
Sundram et al. 2007	Cross-over	Industrial	32 (11/21)	4 wk	Diet	Random order	6.3	3.2	0.43
Chardigny et al. 2008	Parallel	Industrial	40 (19/21)	3 wk	Diet	Random stratified by sex	13.0	5.8	0.20
Motard-Belanger et al. 2008	Cross-over	Industrial	38 (38/0)	4 wk	Diet	Random order	20.8	2.9	0.16
Wanders et al. 2010	Cross-over	Industrial	61 (25/36)	3 wk	Diet	Random order	3.2	7.3	0.20
Desroches et al. 2005	Parallel	ruminant	16 (16/0)	4 wk	Diet	Random	5.9	2.2	0.08
Tholstrup et al. 2006	Parallel	ruminant	42 (42/0)	5 wk	Diet	Random stratified by BMI	0	1.1	0.00
Tricon et al. 2006	Parallel	ruminant	32 (32/0)	6 wk	Diet	Random stratified by age, BMI, triglycerides	3.1	2.31	0.07
Chardigny et al. 2008	Parallel	ruminant	40 (19/21)	3 wk	Diet	Random stratified by sex	13.0	6.6	0.23
Motard-Belanger et al. 2008	Cross-over	ruminant	38 (38/0)	4 wk	Diet	Random order	20.8	2.9	0.23
Motard-Belanger et al. 2008	Cross-over	ruminant	38 (38/0)	4 wk	Diet	Random order	20.8	0.7	−0.10
Berven et al. 2000	Parallel	CLA 50:50	47 (30/17)	12 wk	Supplement	Random (blocks of 2–4)	8.3	1.4	0.25
Benito et al. 2001	Parallel	CLA 50:50	17 (0/17)	9 wk	Supplement	Random	unclear	1.6	0.09
Riserus et al. 2001	Parallel	CLA 50:50	24 (24/0)	4 wk	Supplement	Random	4	1.7	0.35
Smedman and Vessby 2001	Parallel	CLA 50:50	50 (25/25)	12 wk	Supplement	Random	5.7	1.7	0.22
Noone et al. 2002	Parallel	CLA 80:20	51 (18/33)	8 wk	Supplement	Random	0	0.7	0.06
Noone et al. 2002	Parallel	CLA 50:50	51 (18/33)	8 wk	Supplement	Random	0	0.8	0.15
Moloney et al. 2004	Parallel	CLA 50:50	31 (?/?)*	8 wk	Supplement	Random	0	0.9	−0.37
Naumann et al. 2006	Parallel	c9t11CLA	92 (51/41)	13 wk	Supplement	Random	5.4	1.2	0.24
Naumann et al. 2006	Parallel	t10c12CLA	92 (51/41)	13 wk	Supplement	Random	5.4	1,2	0.16
Lambert et al. 2007	Parallel	CLA 50:50	64 (26/38)	12 wk	Supplement	Random	3.1	1.6	0.14
Steck et al. 2007	Parallel	CLA 50:50	48 (13/35)	12 wk	Supplement	Random	12.7	1.3	0.08
Steck et al. 2007	Parallel	CLA 50:50	48 (13/35)	12 wk	Supplement	Random	12.7	2.6	0.15
Iwata et al. 2007	Parallel	CLA 50:50	60 (60/0)	12 wk	Supplement	Random	0	1.4	−0.07
Iwata et al. 2007	Parallel	CLA 50:50	60 (60/0)	12 wk	Supplement	Random	0	2.7	0.11
Gaullier et al. 2007	Parallel	CLA 50:50	105 (21/84)	6 mo	Supplement	Random	21.2	1.4	−0.05
Sluijs et al. 2010	Parallel	CLA 80:20	401 (167/234)	6 mo	Supplement	Random	13.7	1.6	−.0.01
Wanders et al. 2010	Cross-over	CLA 80:20	61 (25/36)	3 wk	Diet	Random order	3.2	8.9	0.29

industrial = industrial trans fatty acid mixture; ruminant =  natural *trans* fatty acids from milk fat;CLA =  conjugated linoleic acid; CLA 50:50 = 50:50 mixture of c9,t11 CLA and t10,c12 CLA; CLA 80:20 = 80:20 mixture of c9,t11 CLA and t10,c12 CLA.

*patients with type 2 diabetes.

**Figure 3:**
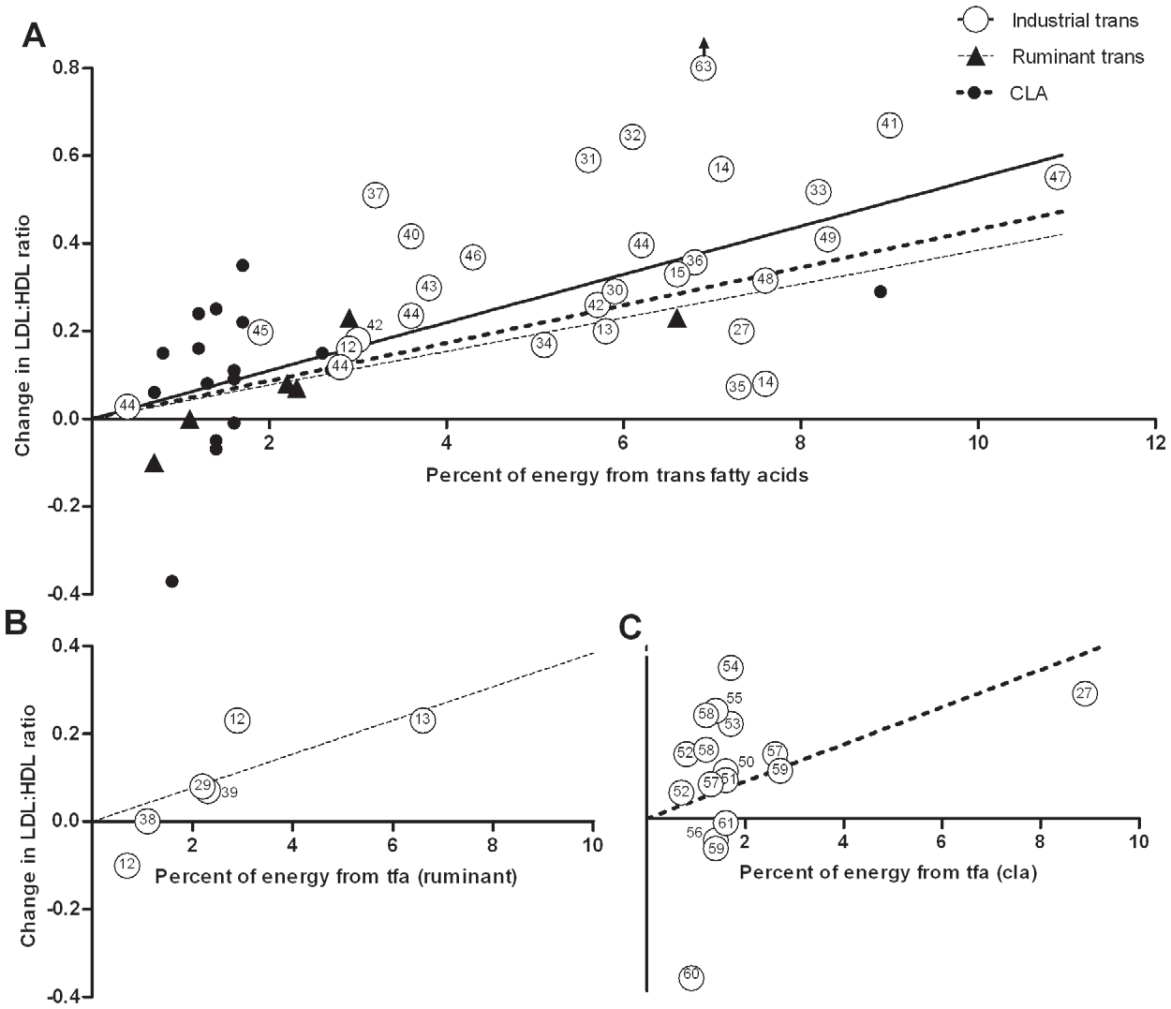
Results of randomized studies of the effects of diets high in industrial *trans* fatty acids (---○○○○○ ---) or ruminant *trans* fatty acids (····▴····) or CLA (▪▪▪•▪▪▪) compared with *cis*-unsaturated fatty acids on the ratio of LDL- tot HDL-cholesterol. a: Results of all studies on the ratio of LDL- to HDL-cholesterol. Results of studies using saturated fatty acids as comparison group [14], [15], [30], [31], [37], [38], [41], [49], [60], [61] and of the Transfact study[13], which compared two sources of trans fatty acids were recalculated to effects relative to isocaloric amounts of *cis* mono-unsaturated fatty acids according to Mensink et al. [26]. To maintain uniformity, we calculated the ratio of LDL to HDL cholesterol from mean LDL and HDL levels, even where ratios had been reported. Numbers indicate reference numbers. Point no. 63 was not included in estimating the regression line because we considered it an outlier. Regression lines were forced through the origin because a zero change in diet should produce a zero change in blood lipids. The black solid line indicates the best-fit regression for industrial *trans* fatty acids (y = 0.055x), the dashed line for ruminant *trans* fatty acids (y = 0.038x) and the grey line for CLA (y = 0.045x). The slopes of the regression lines were not significantly different. b: Results of randomized studies of the effects of diets high in ruminant *trans* fatty acids compared with *cis*-unsaturated fatty acids on the ratio of LDL- to HDL-cholesterol. Results of one study using saturated fatty acids as comparison group [38] and of the Transfact study, which compared two sources of trans fatty acids [13], were recalculated to effects relative to isocaloric amounts of *cis* mono-unsaturated fatty acids according to Mensink et al. [26]. To maintain uniformity, we calculated the ratio of LDL to HDL cholesterol from mean LDL and HDL levels, even where ratios had been reported. Numbers indicate reference numbers. c: Results of randomized studies of the effects of CLA compared with *cis*-unsaturated fatty acids on the ratio of LDL- to HDL-cholesterol. To maintain uniformity, we calculated the ratio of LDL to HDL cholesterol from mean LDL and HDL levels, even where ratios had been reported. Numbers indicate reference numbers. Results of two studies using placebo supplements with a high saturated fat content [60], [61]were recalculated to effects relative to isocaloric amounts of *cis* mono-unsaturated fatty acids according to Mensink et al. [26].

**Figure 4:**
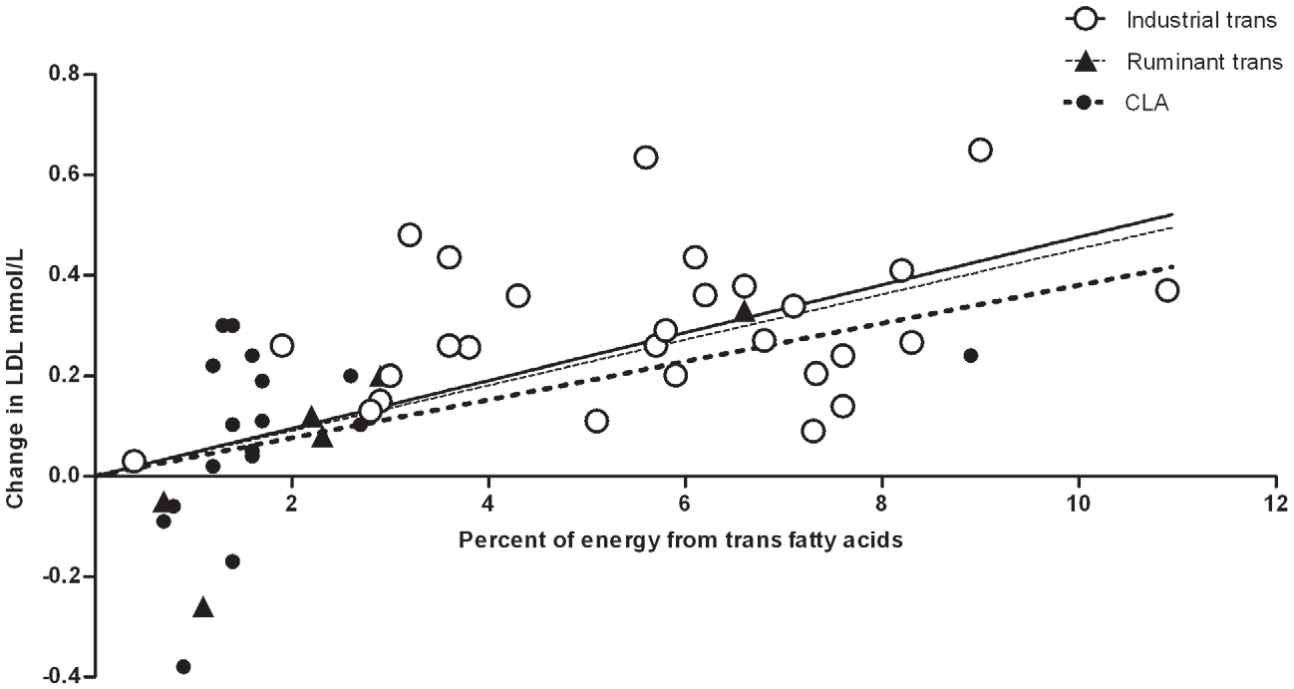
Results of randomized studies of the effects of diets high in industrial *trans* fatty acids (---○○○○○ ---) or ruminant *trans* fatty acids (····▴····) or CLA (▪▪▪•▪▪▪) compared with *cis*-unsaturated fatty acids on LDL cholesterol.

**Figure 5:**
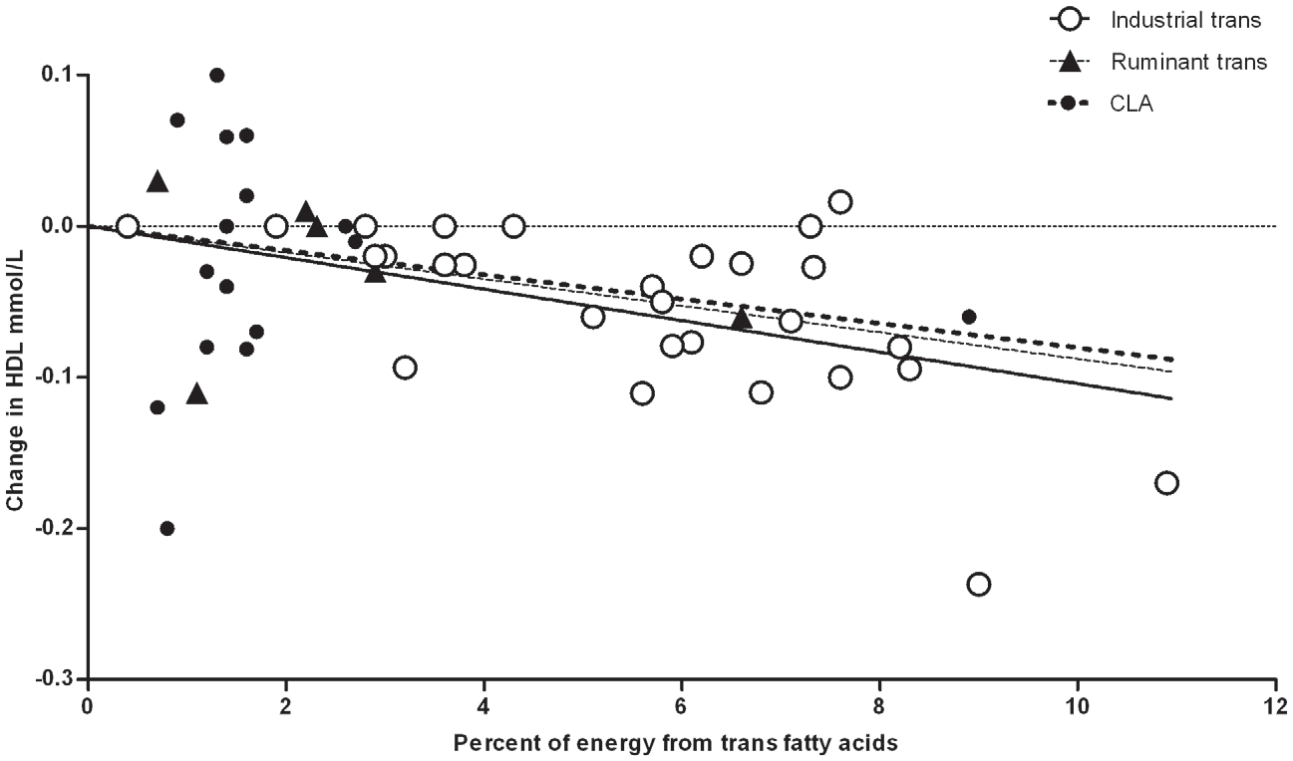
Results of randomized studies of the effects of diets high in industrial *trans* fatty acids (---○○○○○ ---) or ruminant *trans* fatty acids (····▴····) or CLA (▪▪▪•▪▪▪) compared with *cis*-unsaturated fatty acids on HDL cholesterol.

The 23 trials provided 28 data points on the effect of industrial *trans* fatty acids. Linear regression showed that the plasma LDL to HDL ratio increased by 0.055 (95% CI 0.044–0.066) for every % of dietary energy provided by these artificial *trans* fatty acids in the place of *cis*-monounsaturated fat (figure 3a). LDL increased by 0.048 mmol/L (95% CI 0.037 to 0.058; figure 4) and HDL decreased by −0.01 mmol/L (95% CI −0.013 to −0.007) (figure 5) for each % of energy from industrial *trans* fatty acids replacing *cis*-monounsaturates.

The 5 trials provided 6 data points on the effect of ruminant *trans* fatty acids from milk. Linear regression showed that the plasma LDL to HDL ratio increased by 0.038 (95% CI 0.012–0.065) when one % of dietary energy as *cis*-monounsaturated fat is replaced by these natural trans fatty acids (figure 3b). LDL increased by 0.045 mmol/L (95% CI −0.02 to 0.093; figure 4) and HDL decreased by −0.009 mmol/L (−0.025 to 0.007) (figure 5) for each % of energy from animal *trans* fatty acids replacing *cis*-monounsaturates.

The 13 trials provided 17 data points on the effect of CLA supplements. Linear regression showed that the plasma LDL to HDL ratio increased by 0.043 (0.012–0.074) for every % of dietary energy as CLA replacing *cis*-monounsaturated fat (figure 3c). LDL increased by 0.038 mmol/L (95% CI 0.005 to 0.071; figure 4) and HDL decreased by -0.008 mmol/L (−0.023 to 0.007) (figure 5) for each % of energy from CLA replacing *cis*-monounsaturates. We excluded the study of Tricon et al. [62] because of its sequential design. If we included this study using the baseline values as control the slope of the regression line for LDL to HDL changed from 0.043 to 0.041.

The effect of CLA on the LDL to HDL ratio increased to 0.064 per % of energy if we excluded our own study [27] from the regression analysis. This was the only controlled dietary study on CLA, but it used a much higher dose of CLA, namely approximately 20 grams/day as opposed to doses between 1.8 and 6.8 grams/day in the CLA supplement studies. Most studies used supplements with a 50∶50 ratio of *cis*-9, *trans*-11 and *trans*-10, *cis*-12 CLAs. If we excluded the study that investigated these CLA isomers separately [58] and the two interventions that used CLA with an 80∶20 ratio of the *cis*-9, *trans*-11 and *trans*-10, *cis*-12 isomers [27], [52], the effect of CLA on the LDL to HDL ratio became 0.056.

The slope of the regression line for the LDL to HDL ratio was steeper for industrial *trans* fatty acids than for ruminant trans fatty acids or CLA, but the differences between the regression coefficients did not reach any limit of statistical significance. The p-value was p = 0.37 for ruminant versus industrial, p = 0.99 for CLA versus industrial and p = 0.64 for CLA versus ruminant, or p = 0.36 if we excluded our own study [27]. For ruminant *trans* fatty acids the explained variance (R^2^) of effects on the LDL to HDL ratio among the studies included in the regression analysis was 74%. A logarithmic model showed a better fit for the data, with an explained variance of 89%. However, this model did not make biological sense because it predicted that a zero intake of ruminant *trans* fatty acids would cause an infinitely large decrease in the LDL to HDL ratio.

## Discussion

This review provides the first quantitative comparison of the effect of ruminant *trans* fatty acids and CLA with that of industrial *trans* fatty acids on blood lipoproteins in humans. Our analysis shows that all three classes of *trans* fatty acids raise the ratio of LDL to HDL, and therefore, presumably, the risk of coronary heart disease. The effect of ruminant *trans* fatty acids and CLA on the LDL to HDL ratio was less than that of industrial *trans* fatty acids although the difference was not significant. Further studies will be needed to decide whether this difference is real or due to chance.

### Strengths and Limitations

Our search strategy ensured that we included all important studies. We cannot completely exclude the possibility of publication bias. In theory, interests of the sponsoring industry could have prevented publication of studies that showed an unfavorable increase in LDL and/or a decrease in HDL-cholesterol. We have no indications that results have not been published, but if they exist then the adverse effects of ruminant *trans* fatty acids and CLA on blood lipids may have been larger than shown here.

All supplement trials included were performed double-blind, except for one which was single blind [51]. In the dietary studies masking was attempted but was probably incomplete. However, we consider it unlikely that this influenced cholesterol concentrations.

In our calculations we did not take differences in size between the studies into account. This would require standard errors of treatment differences within studies, which generally were not given. We do not think this will affect our estimations much, because individual studies are close to the estimated regression line (figure 3). For the CLA studies this is also not expected to affect the line considerably, because almost all studies had between 50 and 100 participants. Thus, weighing for size is not expected to change the regression line much.

Comparison of the effect of the two CLA isomers – the 9,11 isomers found in milk and supplements, and the 10,12 isomer found only in supplements - is difficult as most studies only investigated a 50∶50 mixture of *cis*-9, *trans*-11 and *trans*-10, *cis*-12 CLA. The studies that investigated either the 80∶20 mixture or pure *cis*-9, *trans*-11 CLA found less of an effect. We cannot exclude that *trans*-10, *cis*-12 CLA raises the LDL to HDL ratio more than *cis*-9, *trans*-11 CLA. However, it is clear that all CLA compounds raise the LDL to HDL ratio.

### Validity of the Models

The first study on industrial *trans* fatty acids and blood lipoproteins in humans [47] reported an adverse effect of *trans* fatty acids, but a high dose not found in regular diets. At the time it was suggested that there was a threshold for this effect and that lower intakes of *trans* fatty acids as found in regular foods had no effect. Later studies showed that this was not the case and that the effect was proportional with the dosage up to very high intakes (figure 3). Mensink et al. [26] showed that effects of saturated and cis- and trans- unsaturated fatty acids on lipoproteins are also linear with dosage. Figures 3b and c suggest that the same holds true for conjugated linoleic acid and ruminant *trans* fatty acids, and that there is no threshold level below which these *trans* fatty acids fail to raise the LDL to HDL ratio.

Figure 3b depicts the dietary studies on ruminant *trans* fatty acids. In this figure only studies on total ruminant *trans* fatty acids are included because there are no dietary studies on isolated vaccenic acid or pure *cis*-9, *trans*-11 CLA. For total ruminant *trans* fatty acids the explained variance (R^2^) was 74%. A logarithmic model showed a better fit with an R^2^ of 89%. However, calculations of explained variance are of limited value because intakes of ruminant *trans* fatty acids did not follow a normal distribution (figure 3b). Besides, this model does not make biological sense because a zero intake would cause an infinitely large decrease in the ratio of LDL to HDL. Furthermore, the logarithmic model was completely driven by a single study that found a decrease in the ratio of LDL to HDL with a small increase in the intake of ruminant *trans* fat. That study was underpowered to convincingly show an effect of this low dose [12]. Therefore, we consider the linear regression line the most parsimonious model for these studies. However, it is based on a limited number of data points and the coefficient may change as more data become available.

For the CLA studies the linear model also seems the most appropriate model. Figure 3c again gives no indication for a threshold below which CLA has no effect.

It was earlier shown that various *trans* 18∶1 isomers (fig 1a; [47], [48]), *trans* isomers of alpha-linolenic acid (fig 1e; [16]) and *trans* C20 and C22 isomers (fig 1f; [14], [15]) raise the LDL to HDL cholesterol ratio. Our present review adds ruminant trans fatty acids and CLA. This suggests that all *trans* fatty acids, including *cis*-9, *trans*-11 CLA, share the same qualitative effect on the LDL to HDL cholesterol ratio in humans (Figure 3a). Although the slopes of the regression lines in figure 3a were not significantly different, we cannot exclude the possibility that small differences in effect exist between *trans* fatty acids from various sources.

A stringent of our conclusion will be provided by an ongoing study at the United States Department of Agriculture [http://clinicaltrials.gov/ct2/show/NCT00942656]. It examines pure vaccenic acid, a *trans* fatty acid not included in our analyses. We predict that 3% of energy from vaccenic acid will significantly raise the LDL to HDL cholesterol ratio by about 0.11 compared to *cis*-monounsaturates. The study also examines 1% of energy from *cis*-9, *trans*-11 conjugated linoleic acid. This should raise the LDL to HDL ratio, but not sufficiently to reach significance. A Swiss study [http://clinicaltrials.gov/ct2/show/NCT00933322] will examine the effect of 2% of energy from *trans* fatty acids in butter. This should raise the LDL to HDL cholesterol ratio by 0.076, but the study may lack the statistical power to pick up this effect.

### Public Health Implications

Most of the *trans* fatty acids in milk and meat consist of vaccenic acid and other *trans*-mono-unsaturated fatty acids similar to those found in partially hydrogenated vegetable oils (figure 1). Our results suggest that all such fatty acids with a double bond in the *trans* configuration raise LDL and lower HDL cholesterol. Removing all such ruminant *trans* fatty acids from the diet would lower the total *trans* fatty acid intake in the United States and Europe by about 0.5% of energy [9], [64] and might therefore reduce cardiovascular disease risk by 1.5 to 6% [5]. Such a specific removal of ruminant *trans* fatty acids from milk and meat is, however, technically not feasible. Our results do reinforce the widespread advice to reduce intake of ruminant fats, because these are also the major source of saturated fatty acids in affluent diets. Recent changes in dairy cattle feeding have led to milk with a lower content of saturated fatty acids and a higher content of *cis*-9, *trans*-11 CLA and other dairy *trans* fats [65]. Our data suggest that the effect of these changes on heart disease risk in consumers of milk and meat fat are at the very least equivocal.

CLA is a minor animal *trans* fatty acid. The effect of dietary CLA on cholesterol will be negligible if we assume that our model is correct. However, intakes from supplements can easily reach 3 grams of CLA a day. This should increase the LDL to HDL cholesterol ratio by 0.050, which would correspond with a 3 to 12% increase in the risk of cardiovascular disease [5].

### Conclusion

Based on this overview we speculate that all fatty acids with one or more bonds in the *trans* configuration raise the ratio of LDL to HDL cholesterol irrespective of their origin or structure. Thus, our results provide an additional argument besides the high content of saturated fatty acids to lower the intake of ruminant animal fats.
