# Correction: Effect of Animal and Industrial *Trans* Fatty Acids on HDL and LDL Cholesterol Levels in Humans – A Quantitative Review

**DOI:** 10.1371/annotation/c4cf3127-89b2-4d58-abf3-ab0746342a90

**Published:** 2010-10-27

**Authors:** Ingeborg A. Brouwer, Anne J. Wanders, Martijn B. Katan

(A) We noted one error and an omission of one relevant paper in our recent review. The difference in the LDL/HDL ratio in the study of Sundram et al. 1997 (reference 63) was 1.06, not 0.75 as listed in Table 1. This error does not impact any of our other data and calculations. After our initial search we excluded one paper [1] that should have been retained, because it meets all our criteria. Sanders et al. [1] reported a randomized cross-over controlled feeding study in which the effect of 10% of dietary energy as industrial trans-monounsaturated fatty acids was compared with the cis-monounsaturated fatty acid oleic acid. Twenty-nine healthy men consumed each diet for 14 days. The LDL/HDL ratio was 0.26 higher on the trans fat diet than on the cis fat diet. Inclusion of this paper produced minor changes in the coefficients of the regression equations. The plasma LDL/HDL ratio now increased by 0.053 (95% CI, 0.042-0.063) instead of 0.055 for each % of energy as industrial trans fatty acids replacing cis unsaturated fatty acids. LDL now increased by 0.045 mmol/L (95% CI, 0.035-0.055) instead of 0.048 mmol/L. The effect on HDL did not change. Inclusion of this study slightly reduced the difference between industrial and ruminant trans fatty acids in the slope of the regression lines for the LDL/HDL ratio. The p-value for the difference between ruminant and industrial trans fatty acids rose from p=0.37 to p=0.42, and the p-value for a difference between CLA and industrial trans fatty acids now became p=0.92. These changes in no way affect the conclusion of our paper, namely that industrial and ruminant trans fatty acids have similar effects on blood lipoprotein concentrations.

1. Sanders TAB, Oakley FR, Crook D, Cooper J, Miller GJ (2003) High intakes of trans monounsaturated fatty acids taken for 2 weeks do not influence procoagulant and fibrinolytic risk markers for CHD in young healthy men. Br J Nutr 89: 767-776.

(B) In addition, some potential competing interests were not declared for Drs. Katan and Brouwer. The authors apologize for this omission and would like to disclose the following information:

Dr. Katan has acted as Supervisor for PhD students whose research was funded by the Wageningen Centre for Food Sciences and its successor, TI Food and Nutrition. This partnership receives funding from the Netherlands Ministry of Economic Affairs, five research organizations (University of Groningen, Wageningen University and Research Centre, Maastricht University, NIZO Food Research, and TNO Quality of Life) and six Dutch food industries. Between 2004 and 2007, Dr. Katan attended scientific symposia organized by Nestlé Research, and expenses related to attendance were paid by Nestlé. Dr. Brouwer was employed by Wageningen University and posted to Wageningen Centre for Food Sciences (see above) for 100% of her time from 1999 to 2005. She then moved to VU University but continued with Wageningen Centre for Food Sciences and its successor TI Food and Nutrition for 40% of her time in 2006 and for 10% in 2007 and the first half of 2008. Her research involved B vitamins and omega-3 fatty acids, and was unconnected with the topic of our present review. In 2004 and 2005, Dr. Brouwer assisted in a study on blood pressure that was supported by a grant from Unilever. 

